# Loops Determine the Mechanical Properties of Mitotic Chromosomes

**DOI:** 10.1371/journal.pone.0029225

**Published:** 2011-12-27

**Authors:** Yang Zhang, Dieter W. Heermann

**Affiliations:** Institute for Theoretical Physics, Heidelberg University, Heidelberg, Germany; Dalhousie University, Canada

## Abstract

We introduce a new polymer model for mitotic chromosomes. The key assumption of the model is the ability of the chromatin fibre to cross-link to itself due to binding proteins. These protein-chromatin interactions are included by a probabilistic and dynamic mechanism. The hypothesis is motivated by the observation of high repulsive forces between ring polymers. We performed computer simulations to validate our model. Our results show that the presence of loops leads to a tight compaction and contributes significantly to the bending rigidity of chromosomes. Moreover, our qualitative prediction of the force elongation behaviour is close to experimental findings. The Dynamic Loop Model presented here indicates that the internal structure of mitotic chromosomes is based on self-organization of the chromatin fibre rather than attachment of chromatin to a protein scaffold. It also shows that the number and size of loops have a strong influence on the mechanical properties. We suggest that changes in the mechanical characteristics of chromosomes in different stages of the cell cycle, for example, can be explained by an altered internal loop structure.

## Introduction

During mitosis the dispersed interphase chromosomes undergo a transition into rigid, tightly compacted objects. This condensation mechanism and the inner structure of the chromosomes in this phase has been the target of many studies so far. The lengthwise compaction ratio of DNA in mammalian metaphase chromosomes is of the order of 10 000–20 000 [1]. On the lowest folding level, double-helical DNA wraps around histone octamers and thus forms nucleosomes about every 200 base pairs [2]. In a next step the coiling of this 10 nm thick beads-on-a-string fibre into a 30 nm thick filament was suggested [3]–[5]. These first two levels of folding account for a 40 fold compaction of the naked DNA. However, the existence of the 30 nm fibre is still under debate. The higher order folding motifs that are responsible for the remaining approx. 500 fold compaction still remain largely unknown [6], [7].

Many models have been put forward for the description of the chromatin structure in mitosis, including radial loop models, hierarchical folding models and network models [8]. In an early model Bak et al [9] suggested a helical folding of a 400 nm thick chromatin fibre. The composition of the chromosome of a thin fibre of 200–300 nm in diameter was also proposed by Sedat and Manuelidis [10]. Most textbooks feature the radial loop model which is based on histone-depletion experiments. It assumes the chromosome shape to be essentially governed by an axial non-histone protein scaffold to which chromatin loops are attached [11], [12]. Condensins and Topoisomerase II were found to be the main components of the protein core and are therefore the main candidates for the driving forces of the condensation [13], [14]. However the radial loop model has been put more and more into question. Different experiments report that Topoisomerase II and condensins are highly mobile within mitotic chromosomes [15], [16]. Kireeva et al [17] showed that axial staining of condensins cannot be seen until late prophase when considerable condensation has already taken place. Instead of an axial protein scaffold, the authors suggest a hierarchical folding of the chromatin fibre. These kind of models predict different folding levels from the 30 nm fibre into the 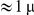
m thick chromosome. Possible folding levels are a 100–130 nm fibre and subunits in the size of 250 nm [10], [18], [19].

Another approach to the analysis of mitotic chromosome structure are micromechanical manipulation experiments which target the elastic properties of chromosomes [20]. Human chromosomes and chromosomes from newt lung cells were found to be very elastic objects which can be stretched to several times of their native length [21], [22]. Houchmandzadeh and Dimitrov measured the bending rigidity and the stretching stiffness of single in vitro assembled chromatids from Xenopus egg extract. They found the chromatids to be very flexible objects with the persistence length being only a few times the thickness of the chromosomes. For small extensions the authors reported a linear force-elongation behaviour and reversible deformability. Furthermore chromosomes were extensible up to 100 times their native length with a force plateau being observed at relative extensions larger than 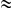 15 [23]. This kind of elastic response was also confirmed for chromosomes from newt lung cells. The chromosomes showed reversible extension up to three times of their native length. For intermediate extensions, hysteresis was observed and for long extensions beyond 30 times of the native length, the force-extension curve decreased to a plateau [24]. Further experiments by Poirier and Marko [25] targeted the force-relaxation behaviour of stretched chromosomes, especially during nuclease digestion. The authors concluded that mitotic chromosomes do not have a mechanically contiguous protein scaffold but rather proposed a network model, where the 30 nm chromatin fibre is cross-linked to itself by binding proteins [26]. While results on the stretching stiffness agree widely for chromosomes of different species, this is not the case for the bending rigidity. As described above, Xenopus egg extract chromatids were reported to be very flexible [7], [23]. However, experiments on in vitro and in vivo assembled chromosomes from other animals yielded much higher rigidities with persistence lengths being much larger than the length of the chromosomes themselves [27], [28]. Recent investigations showed that human mitotic chromosomes have very similar mechanical properties to mitotic newt chromosomes and thus likely smiliar structures, too [29].

All studies on mitotic chromosome structure indicate that chromatin loops play an important role in its organization. Loops can compact the chromatin fibre and be in part responsible for the mechanical properties. Especially the size and the number of loops were suggested to be closely connected to them [30], [31]. Moreover, there is evidence that looping of the chromatin fibre is crucial for chromosome compaction during all stages of the cell cycle. FISH experiments and new 3C/4C/5C/HiC experiments showed that loops of all length scales can be found in the interphase chromosome, possibly connected to transcriptional activity and genome function [32]–[36]. The Random Loop Model and its further development, the Dynamic Loop Model, assume dynamic formation of loops on all length scales. They predict the confined folding of the chromosome without spatial constraints [37]–[39]. However, in contrast to the interphase, the chromatin fibre does not show long range interactions between distant segments in mitosis. Estimates for loop sizes here are in the range of 20 to 90 kb [11], [25]. Marko [40] pointed out that local coiling of a polymer along its length while long-range cross-linking is absent can be responsible for a lengthwise condensation.

In this work we investigate how the formation of loops can account for the condensation and mechanical properties of chromosomes during mitosis. Polymer rings have been found to repel each other much stronger than linear polymers [41]. Therefore, looping alone can already be responsible for a considerable stiffening up of the chromosome. However, our model does not impose an ordered structure on the chromatin fibre. Rather, cross-links and thus loops are formed upon collisions of fibre segments. Condensins and Topoisomerase II were suggested to be responsible for the cross-linking of chromatin during mitosis [1]. The probabilistic nature of our model, where loops form and dissolve dynamically, can account for the mobility of these proteins within the chromosome.

For the mitotic chromosomes we assume a restricted interaction range for the formation of cross-links in order to achieve a lengthwise compaction of the chromatin fiber. The restricted interaction range of the cross-links is motivated by the geometrical shape of mitotic chromosomes, which appear to be rod-like and thus very different to the more spherical shapes of chromosome territories in interphase. On the other hand, a strong compaction in length especially in eukaryotic mitotic chromosomes can be observed. Therefore, the model assumes a short-ranged folding of the chromatin fiber that results in a length-compaction and condensation of the fiber into rigid rods. This kind of folding also guarantees that genes are aligned linearly along the mitotic chromosome. Essentially, the coiling of the chromatin fiber can be seen as the folding of a thin fiber into a thick fiber.

Our results suggest that mechanical properties can be explained by self-organization of the chromatin fibre without the need of any axial protein scaffold. With this dynamic formation of loops, the resulting structure of the chromatin fibre is similar to a chromatin network. Moreover, our model can be seen in the context of a hierarchical folding model.

## Results

### Dynamic Loop Model for mitotic chromosomes

Condensins and Topoisomerase II are presumably the proteins that establish cross-links of chromatin in mitotic chromosomes [13], [14]. Christensen et al [15] found Topoisomerase II to be mobile in human mitotic chromosomes. High mobility of condensin I in Drosophila metaphase chromosomes was reported by Oliveira et al [16]. Hence, the loop structure of mitotic chromosomes is not fixed but rather subject to fluctuations in loop sizes and positions of the loops. However, the complex dynamics of proteins and their interactions with the chromatin fibre are too complicated to be modeled in detail on the scale of a complete chromatid. Coarse grained approaches are therefore used to model the chromatids.

We present a model, where the cross-linking due to Topoisomerase II and condensins is incorporated by a dynamical looping mechanism of the fibre. This mechanism consists of the ability of distant fibre segments to form cross-links when they come into physical proximity of each other by diffusion. The shape of mitotic chromosomes is rod-like, as opposed to the more spherical shape of interphase chromosomes. Bohn et al [39] have shown before that long-range interactions unevitably lead to spherical shaped objects if the number of cross-links is high, which it has to be in the case of highly condensed mitotic chromosomes. On the other hand, short ranged interactions and the lack of long-range interactions at the same time were discussed to be responsible for a lengthwise condensation of the chromatin fiber [40]. Therefore, in this model for mitotic chromosomes, we included a restriction for the interaction range of the chromatin fiber and thus for the maximum loop size. Below this limit, all loop sizes are equally possible.

Such a lengthwise condensation also accounts for the fact that genes are aligned linearly in mitotic chromosomes, whereas long-range interactions can easily lead to mixing of distant chromosomal parts, bringing genomically distant genes into physical proximity of each other. A restriction of the length of loops is also consistent with experimental observations which do not give any indications for the existence of long-range interactions in mitotic chromosomes. Consequently, all proposed models for the folding of mitotic chromosomes implicitly include restricted interaction ranges with the estimates for loop sizes ranging from 20 kb to 90 kb.

Additionally, in order to mimic the dynamics and mobility of the involved proteins, the cross-links have limited lifetimes, after which they dissolve again. The two important parameters of the model are therefore the restriction on the interaction range, the cutoff length 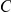 which determines the maximum size of loops, and the number of loops divided by the number of statistical segments that the chromatin fibre consists of, the loop concentration 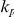. A value of 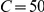 means that segments can only form cross-links if they are separated by no more than 50 statistical segments. A value of 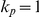 means that there is on average one cross-link and thus one loop per statistical segment.

The dynamic crosslinking mechanism in our model implicitly mimicks the presence of binding proteins like condensins in the surrounding solvent of the chromatin fibre. The cutoff length for the size of loops accounts for the fact that long-range interactions are not present in mitotic chromosomes. However, we do not wish to imply that proteins like condensins would not be able to crosslink two chromatin regions that are genomically far away. Our presented model does not include explicit binding mechanisms of condensins and other proteins to chromatin. Instead, we suggest that condensins and Topoisomerase II cause a local folding up of the chromatin fiber leading to the formation of a thicker and strongly compacted filament. The emerging chromatin-solvent interfase following this compaction could then be a reason for the prohibition of long range interactions within mitotic chromosomes. The cutoff length is meant to account implicitly for such phenomenons which prevent the formation of large loops.

One of the major questions addressed in this work is whether this dynamic looping mechanism could be responsible for the condensation of the chromatin fibre into the mitotic chromosome. Furthermore, it was shown experimentally that mitotic chromosomes have high bending rigidities while still being very elastic. The other important question that is addressed here is to which extent entropic effects invoked by the formation of chromatin loops can account for the mechanical properties of mitotic chromosomes.

### Dynamic looping mechanism promotes condensation into rigid objects

**Figure 1:**
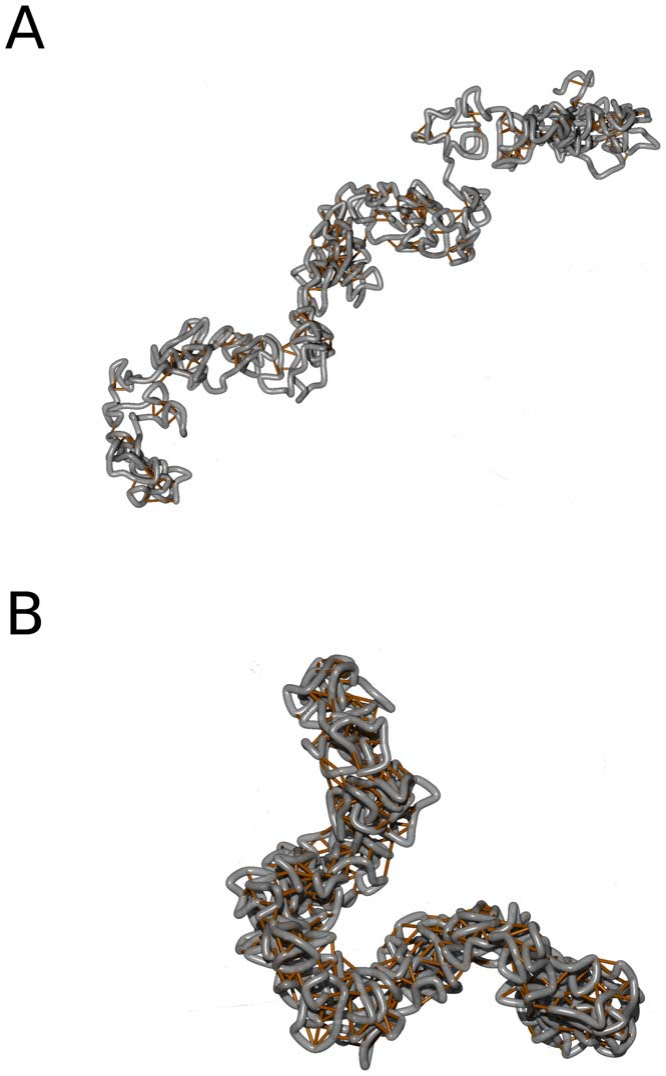
Examples for model chromatids with different parameter sets. The grey tube represents the chromatin fibre. The orange sticks visualize the cross-links between distant fibre segments. Each chain embodies a single chromatid. The chromatin fibre in both examples consisted of 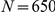 segments. The cutoff length for the loop sizes is 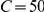. It means that fibre segments which are separated by a genomic distance greater than 50 monomers cannot form an additional bond. **A.** In this example, the mean loop concentration is 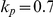. At these low loop concentrations the conformations are non-homogeneous. Rather, a2 formation of blobs can be observed in regions with many cross-links. These regions are connected by fibre section with no or only few loops. **B.** When the loop concentration is high enough, a condensation of the chromatin fibre into thick, homogeneous rods can be observed. In this configuration the loop concentration is 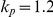. Cross-links are distributed homogeneously along the chain.

We analysed the shape and morphology of the model chromatids. In Figure 1 typical conformations for different parameter configurations can be seen. When no loops are present, the fibre behaves like an ordinary self-avoiding walk. For low loop concentrations, cross-links at different positions along the chromatin fibre are formed. In these regions a compaction and a formation of blobs can be observed. These blobs are connected by fibre sections with no loops. However, when the settings for the looping probability and the mean loop lifetime are increased, the chromatin fibre condenses into a thicker, rod-like filament. The structure then resembles a flexible rod and is homogeneous along its contour. Thus, for high loop concentrations, the Dynamic Loop Model produces coiled fibres with a strong resemblance to mitotic chromatids.

We used coarse grained polymer chains consisting of up to 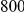 segments to represent the underlying chromatin fibre. Assuming a DNA content of approx. 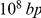 in the chromosome we obtain the size of one statistical segment to be approx. 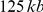. We investigated configurations with loop concentrations up to 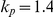. We observed that a value of at least 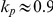 is needed for condensation of the fibre into a rod that resembles a mitotic chromatid. A value of 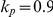, which means on average 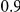 cross-links per statistical segment, would thus correspond to one cross-link every approx. 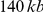. Likewise, 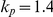 would mean one cross-link every approx. 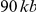.

**Figure 2:**
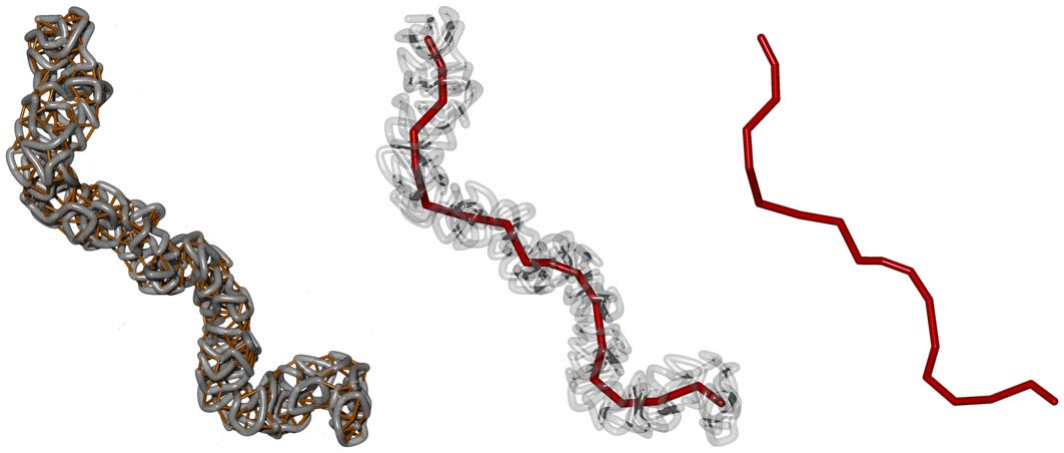
Visualization of the backbone for a model chromatid. In order to analyse the shape and the mechanical properties of the condensed rods, it is necessary to calculate backbones which represent the alignment of the model chromatids. These backbones are obtained by a coarse graining method that is applied to each single conformation. The original polymer chain is divided into 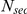 sections that contain 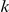 statistical segments each and the center of masses of each section is calculated. The new chain consists of the center of masses of the sections. The degree of coarse graining, characterized by the parameter 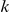 is an important parameter in this method. It has to be chosen correctly in order to guarantee that the backbone truly represents the alignment of the chromatid.

In order to analyse the shape and mechanical properties we calculate backbones which represent the alignment of the model chromatids. Each backbone can be seen as a coarse grained polymer that describes the large scale properties of the model chromatid without the details of the coiling on the local scale. Figure 2 illustrates this fact. The backbones are used to estimate the geometrical properties and the directional correlation between different segments of the chromatids. The mean chromatid lengths are calculated and compared for different settings of cutoff length 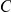 and loop concentration 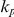. When the maximum loop size 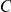 is increased, the length of the rod decreases as large loops condense the fibre more efficiently than small loops. The compaction is also tighter when the mean loop concentration 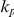 is higher. Therefore, the chromatid length decreases with higher loop concentrations, too.

**Figure 3:**
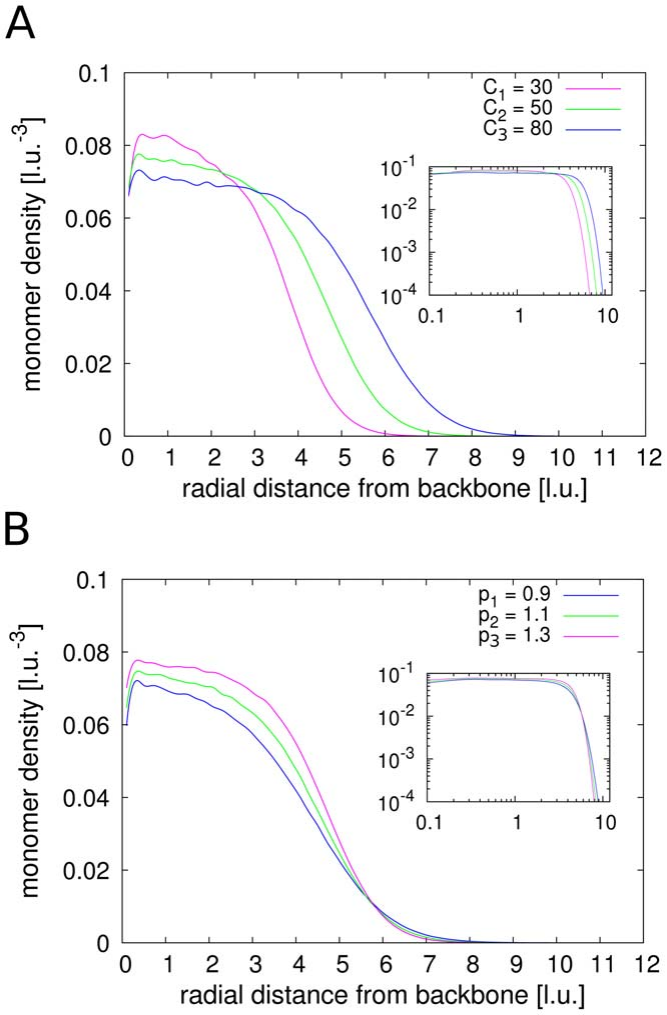
Mean radial monomer density functions 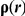 of different configurations. **A.** Larger cutoff sizes 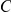 give thicker model chromatids but smaller densities and lengthwise compaction ratios. **B.** The number of loops has only small influence on the chromatid thickness. However, chromatids with more loops show tighter compactions and hence higher monomer densities.

The estimation of the chromatid thickness involves the calculation of the average radial monomer density functions. Radial in this case means perpendicular to the calculated backbone. Figure 3 shows radial monomer density functions and their dependency on the cutoff length 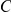 and the loop concentration 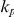. At the central axis the density has a minimum, but increases quickly with the distance from the backbone, until a plateau area is reached. This broad plateau is then followed by the expected decay for large distances. The drop off at the backbone indicates that the fibre tends to form rings around a central axis and is roughly organized in a helical-like structure. However the organization is much more complex than a simple helix. The broad plateau region suggests that the chromatid is radially homogeneous on a large scale.

**Figure 4:**
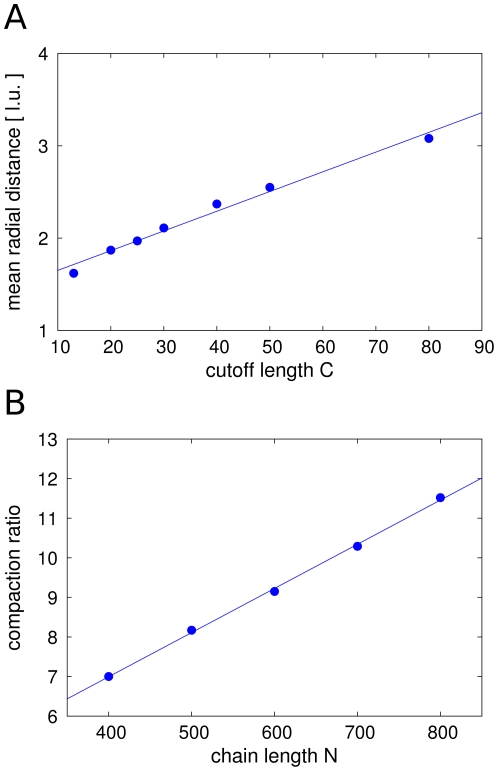
Influence of the parameter settings on the spatial dimensions. **A.** A linear relationship between the average distance of monomers from the backbone and the cutoff length is found. Together with the observed drop off of the monomer density at the central axis we conclude that the fibre coils around the backbone in a helical-like folding manner. However this gives just a general tendency and the exact structure is much more complicated. **B.** Interestingly we can see that at constant values for 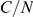 the lengthwise compaction ratio still increases linearly with the chain length 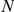. In this example 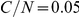 and the mean loop concentration is 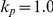. When extrapolating the linear curve to a compaction ratio of 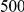-fold, a polymer with 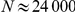 statistical segments and cutoff length 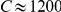 would be needed.

Next we look at the dependency of the density and the width of the model chromatids on the parameters 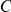 and 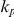. As expected, larger cutoff lengths result in a more extended plateau and thus thicker chromatids. On the other hand the number of cross-links has only little influence on the thickness. For the same cutoff length, conformations with more cross-links only yield higher monomer densities but have the same widths as conformations with fewer cross-links. The dependency of the chromosome thickness on the cutoff length 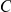 is displayed in Figure 4. A linear behaviour can be observed.

We used many different parameter settings 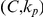 to investigate how they influence the geometry of the resulting model chromosome. To illustrate that the model chromosomes do match the geometry of real chromosomes, for example human metaphase chromosomes, we consider the result of a setup with 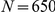 statistical segments, a cutoff length 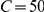 and a loop concentration of 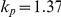. Assuming a lattice constant of 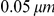, the mean length of the model chromatid would then be 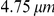 and the thickness would be 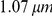, corresponding to a cross-section of 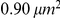. This example demonstrates that our model chromosomes have indeed the dimensions of real chromosomes.

Our results for the geometry of the model chromatids show that the Dynamic Loop Model covers a broad range of different geometries, depending on the parameters 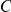 and 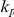. When the maximum loop size is higher, the chromatid is thicker and shorter. On the other hand, the mean loop concentration influences the compaction and length of the chromatid but not the thickness. Therefore, the loop structure, the number and size of loops, obviously plays an important role for the shape of chromosomes. In Table 1 an overview of results on the length and width of model chromatids is given. The length-to-width ratios match those of natural mitotic chromosomes in different stages of mitosis.

**Table 1:** Excerpt of results for size and persistence length of chromosomes.

N	C	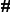 loops/N	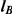 [l.u.]	thickness 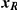 [l.u.]	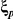 [l.u.]
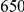	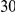	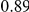	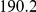	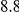	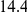
		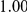	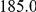	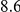	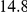
		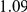	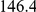	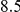	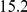
		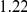	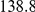	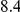	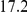
	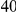	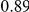	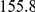	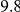	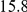
		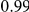	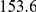	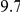	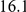
		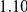	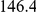	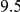	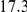
		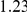	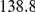	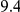	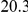
	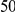	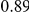	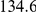	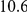	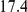
		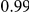	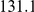	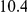	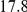
		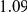	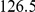	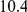	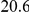
		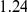	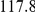	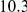	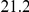
		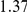	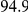	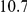	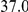
	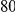	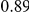	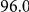	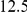	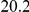
		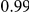	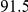	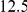	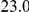
		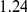	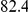	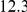	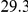

### Presence of loops enhances the bending rigidity due to entropic repulsion

**Figure 5:**
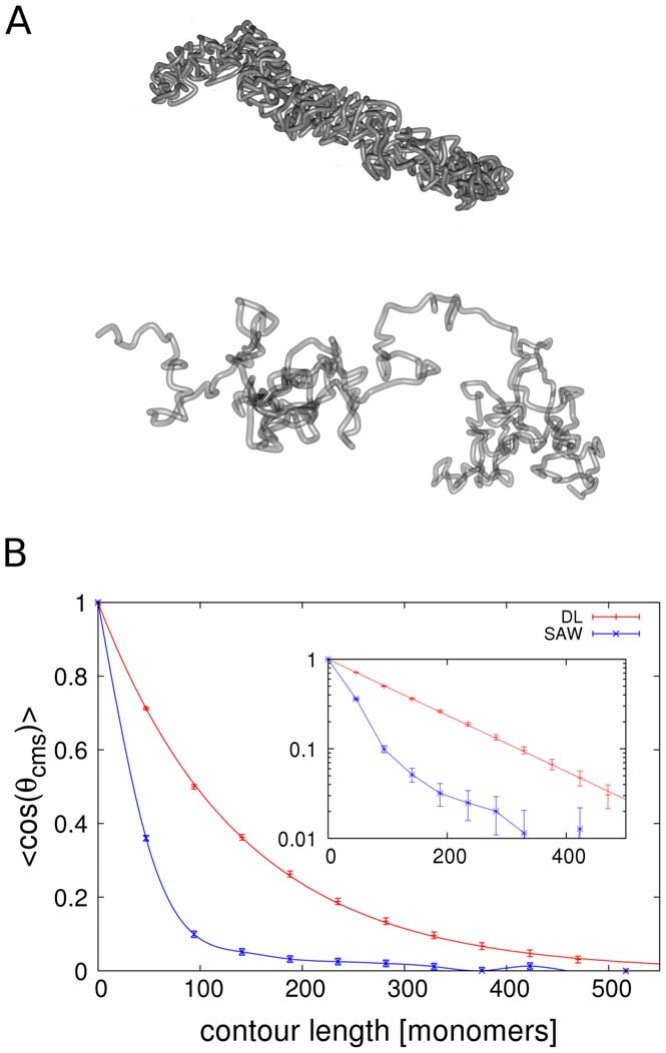
Comparison between Dynamic Loop Model and self-avoiding walk. **A.** The upper conformation is a Dynamic Loop Model chromatid with 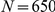, cutoff size 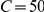 and mean loop concentration 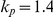. For comparison, a conformation without loops with the same chain length 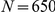 is shown below. **B.** For both, self-avoiding walk and Dynamic Loop Model the coarse-graining method is applied and the directional correlation is calculated. The same degree of coarse-graining is used for both models. The figure shows an exponential decay of the directional correlation function of the Dynamic Loop Model, while the the self-avoiding walk does not show this behaviour. Most importantly, the Dynamic Loop Model chromatid is much stiffer than the self-avoiding walk. This shows that the entropic repulsion of the chromatin loops that are generated by the cross-linking mechanism leads to a considerable stiffening up. Error bars represent the standard error of the sampled conformations.

The analysis of mechanical properties, especially the bending rigidity and the elastic response have been important parts in the experimental examination of mitotic chromosomes. Therefore, we analyse results from our model and from models without loops such as the self-avoiding walk with respect to the directional correlation using the calculated backbones. Figure 5 shows a comparison between the mean directional correlation of both models when the same degree of coarse graining for the calculation of the backbone is used. The directional correlation function for the Dynamic Loop Model shows an exponentially decaying relationship with the separating genomic distance. Therefore the backbone of the model chromatid behaves like a worm-like chain on this length scale. This result is consistent with experimental findings of Houchmandzadeh and Dimitrov [23] who found chromatids from in vitro assembled Xenopus laevis egg extract to show an exponentially decaying mean directional correlation for one order of magnitude. Furthermore, when compared to a self-avoiding walk, the Dynamic Loop Model polymer has a much higher bending rigidity. This is a very important finding as it shows that simply the existence of loops enhances the bending rigidity of the chromatin fibre. The entropic repulsion between polymer rings is responsible for this observation. In the presence of a large number of rings within the chain as in the case of the Dynamic Loop Model, bending of the chromatid will reduce the distance between closely aligned loops. Hence the energy required to bend the chromatid is higher than in the case where no loops are present, leading to an enhanced stiffness of the filament.

However, it should be noted that already the calculation of a backbone for the self-avoiding walk is not meaningful since the conformations of self-avoiding walks do not have the shape of mitotic chromosomes. On the other hand, the rescaling for the Dynamic Loop Model generates worm-like backbones which truly represent the overall alignment of the model chromatid.

**Figure 6:**
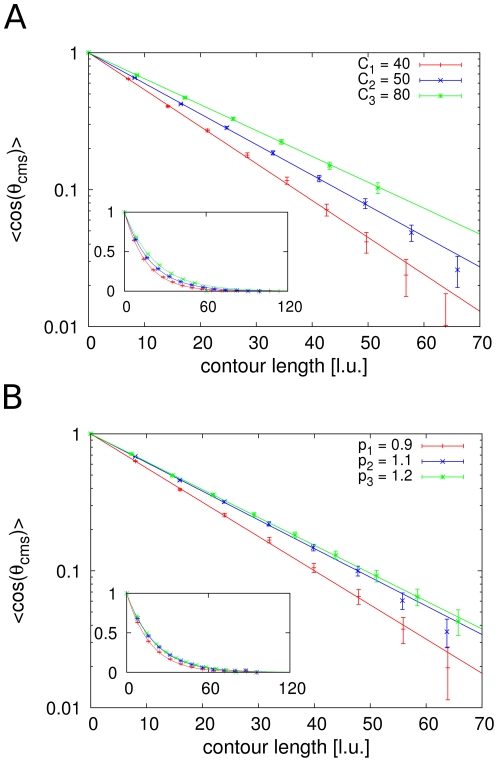
Directional correlation functions for model chromatids with varying parameters. The chain length for all configurations is 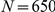. Error bars represent the standard error. **A.** For fixed mean loop concentration 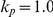 we can see that the stiffness increases with the cutoff length 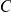. Larger cutoff lengths result in thicker chromosomes and in turn less flexibility. **B.** Shown are results for 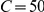 and different mean loop concentrations. An increased number of cross-links is associated with a more densly packed chromosome. Thus, the distance between loops is decreased and the repulsive forces between them are stronger. Consequently higher bending rigidities are obtained.

Simulations with different cutoff lengths and loop concentrations show that the bending rigidity is very sensitive to both parameters. Directional correlation functions for different values of cutoff length and loop concentration can be seen in Figure 6. Increasing the cutoff length results in a higher mean loop size. This in turn leads to chromatids with larger thickness and thus reduced flexibility of the filament. For a homogeneous cylinder, the bending rigidity is proportional to the fourth power of the radius [42]. Furthermore, large loops within the chromatin fibre tie parts of the fibre together which would normally be farther apart. This tightening also contributes to the enhanced stiffness. Consequently, the flexibility of a chromatid with higher cutoff length is reduced compared to a chromatid with smaller cutoff length. The bending rigidity is also influenced by the number of loops within the polymer. Higher mean loop concentrations 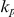 are associated with stronger compactions of the chromatids. Hence, the loops or chromatin rings are spaced closer to each other increasing the entropic repulsive forces between them. Bending becomes therefore even more energy consuming.

**Figure 7:**
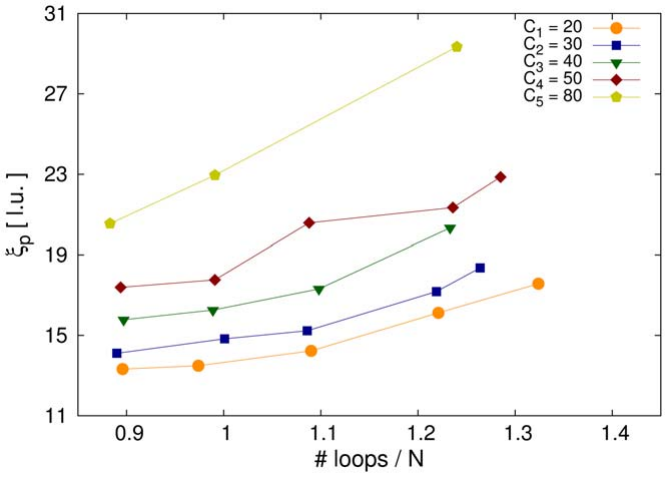
Persistence length in dependency of the cutoff length 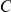 and the mean loop concentration 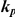. The persistence length and thus the bending rigidity increases with higher cutoff length and higher mean loop concentration. However, no simple dependency can be derived from the results. As the internal structure of the model chromatids is complex, the persistence length also has a complicated relation to the parameters.

The persistence lengths for model chromatids with different parameters are shown in Figure 7. Clearly the bending rigidity increases with the number of loops in the chain and also with higher cutoff lengths. We find typical values of the persistence length to be in the range between 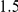 to 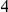 times the diameter of the chromatid, depending on the parameter settings. This is in good agreement with results on mitotic chromatids from Xenopus egg extract [23].

### Variations in size and number of loops evoke different elastic responses

Measuring the elastic response is one possibility to study the internal structure of mitotic chromosomes. Simulations of model chromatids under an external pulling force are done to examine their elasticity. In the pulling simulations, model chromosomes are first subjected to the Monte Carlo algorithm of the Dynamic Loop Model until they are fully condensed. Then a constant pulling force 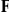 is applied to the chromatid ends directed along the end-to-end vector. A corresponding pulling energy 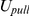 is added to the energy of the conformation. Conformations are then sampled from the equilibrium distribution including the additional pulling potential. Thus, the pulling can be viewed as adiabatic. For fixed parameter sets, we analyse the mean end-to-end distances of the model chromatids at different pulling forces and calculate the mean relative extensions 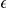.

We have to point out that in our coarse grained model no additional potential between the segments exist apart from the dynamic cross-linking mechanism. Furthermore, the stretching in the model is performed in equilibrium. The stretching forces in our model are therefore much smaller than the forces that are measured in experiments. Assuming a lattice constant of 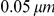 in our model, the forces are in the range of 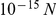 which is several orders of magnitude smaller than forces measured in micromechanical experiments on mitotic chromosomes. However, using this kind of coarse grained modeling it is possible to make qualitative tests and predictions.

**Figure 8:**
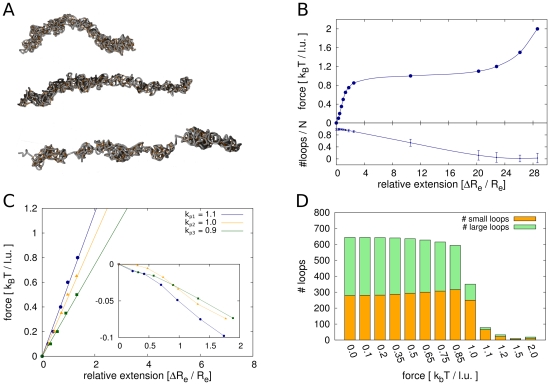
Simulation of pulling of the model chromatids. **A.** Model chromatids at different pulling forces. For small elongations the chromatid is stretched but the total number of cross-links does not change. For higher elongation the number of cross-links decreases rapidly and the chromatid becomes inhomogeneous. **B.** Detailed look at the force elongation curve for the configuration 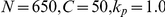. In the range of extensions up to two times of the native length, a linear dependency can be observed, where the total number of cross-links remains nearly constant. For higher extensions a force plateau is reached. Here the number of cross-links decreases and the chromatid is unfolded rapidly. This region corresponds to a decondensation region. **C.** The slope of the force elongation curve in the linear part depends strongly on the mean loop concentration. Here we show results for 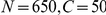 and three different values for 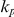. The force modulus for configurations with mean loop concentration 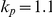 is more than double than the modulus for configurations with 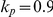. Hence we conclude that different elastic responses can be explained by altered loop structures. The inset shows the relative change in chromatid thickness against the relative extension. Similar Poisson's ratios are obtained for the different configurations. The values are in the range 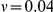 and 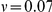 and therefore close to experimental findings. **D.** Although the total number of loops is constant in the linear region, there are changes in the loop structure. Shown are results for 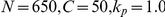. The loop domains are reorganized due to the pulling force, with the proportion of small loops (size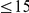) increasing at the expense of the number of large loops (size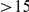). As the thickness is essentially determined by the size of the loops this finding indicates that chromosome width decreases in this area which is consistent with experimental results.

The elastic response of the model chromatids shows different domains. Furthermore, the size and number of loops play a crucial role for the elasticity. Force elongation curves for different settings of cutoff length 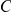 and loop concentration 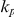 can be seen in Figure 8. For relative extensions of up to twice the native length of the model chromatid, we observe a linear relationship between force and relative extension. This means that in this region the chromosome has the elasticity of a homogeneous, elastic material. Such a behaviour of mitotic chromosomes was found in numerous experiments [23], [24], [29]. In this region no significant decrease in the total number of loops can be seen. The chromatid is stretched but its looping mechanism still efficiently cross-links different chromatin segments. However, the analysis of the loop size distribution shows that although the total number of loops does not change, there is a reorganization of the loop domains. The number of small loops increases while the number of large loops decreases, hence there is a shift from large to small loops. Apparently, when only small forces are applied, the formation of larger loops is inhibited because distant chromatin segments are pulled apart. However on the local scale the looping mechanism is still intact and thus more small loops are formed, keeping the total number of loops constant.

At larger pulling forces the loop formation on all scales is inhibited. The formation of large loops is still obstructed stronger than the formation of small ones. The force extension curve is very flat in this region and resembles a plateau. At this strength, the applied force destroys the cross-linked structure of the polymer. The region of 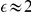 to approx. 20 times elongation of the native length can be characterized as the decondensation domain of the model chromatid. In this domain the internal structure of tightly condensed loops is destroyed. The slope of the force-extension curve is very low in this region and a small increase of the force results in a vast stretching of the chromatid. Such force plateaus were also found in the experiments, although at larger relative extensions [22], [24].

We evaluate the slope of the force extension curves in the linear domain from relative extensions of 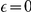 to 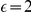 for several different parameter sets. The slope is then used to calculate Young's modulus that characterizes the elasticity of an elastic material. Almagro et al [7] showed that mitotic chromosomes do not have a homogeneous elasticity but that rather different segments show different elastic moduli. Furthermore the elastic behaviour of mitotic chromosomes can be changed by exogenously added agents, such as trypsin, proteinase K or Topoisomerase I and II [43], [44]. These alterations of the mechanical properties were suggested to be related to changes in the internal chromatin structure for example by reducing the number of protein cross-linkers.

Results of our simulations on stretched chromosomes show that the elasticity is highly dependent on the mean loop concentration. We performed simulations with the same cutoff length 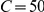 for polymers consisting of 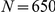 monomers, but different loop concentrations. The results show that when the mean loop concentration is increased from 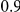 to 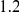, the Young's modulus of the model chromatid increases by a factor of two. Tighter compaction is thus associated with a strongly decreased elastic response. This result is plausible as more cross-links within the fibre means that segments are glued to each other more efficiently and hence are harder to be stretched by an external force. Therefore, the loop formation is also responsible for the elastic response and the number of loops controls the stretching stiffness of the chromatid. We calculated the bending moduli that would be associated with the obtained Young's moduli if chromatids were cylinders made of a homogeneous material. The calculated bending moduli are three to five times smaller than the ones obtained from direct measurement of the flexibility. Table 2 gives an overview over results obtained from both, elasticity measurement and direct measurement of bending fluctuations.

**Table 2:** Comparison for bending stiffness from direct measurement and calculation using elastic response.

N	C	# loops/N	*B* measured [ 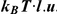 ]	*B* calculated [ 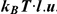 ]
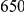	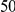	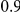	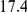	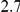
		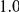	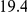	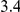
		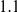	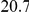	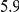
	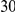	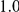	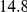	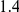
		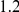	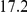	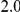

Estimation of the thickness of elongated chromatids show that the widths decrease when the chromatids are pulled. This is consistent with the observed shift of the loop size distribution from larger to smaller loops and the decrease of the mean loop size. We calculated Poisson's ratio to quantify this finding. The inset in Figure 8C shows the relative change in width to the relative change in length of the chromosomes. The relationship is not linear from the start but rather converges to a linear curve. The Poisson's ratio is determined by fitting the linear region of the curves. Experimental studies by Poirier et al [24] resulted in a Poisson's ratio of 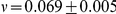 for newt lung cells. For our studies, different Poisson's ratios were obtained for different mean loop concentrations. With values between 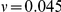 and 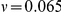, our results are in the same range as the experimental findings.

## Discussion

In this work we used a coarse grained polymer model to investigate if the condensation during mitosis can be understood by a probabilistic, locally restricted cross-linking mechanism of the chromatin fibre. We showed that this mechanism results in a tight compaction of the chromosome. The restriction of the loop sizes by a cutoff length in our model implicitly describes the fact that long range interactions cannot be formed in mitosis while the dynamical formation and dissolution of crosslinks implicitly accounts for the dynamics of the binding proteins in the surround solvent.

Although we do not describe explicitly the binding of proteins to DNA at special binding sites and do not want to state that condensins or Topoisomerase II could not link distant segments of chromatin to each other, we believe that it cannot be excluded that there could be principles that allow binding proteins to distinguish between different chromosomal regions. We believe that the cohesion of sister chromatids can serve as an good example. It seems that cohesin proteins have the ability to distinguish between the chromatin strands of the sister chromatids. Therefore, in the same way, condensins could have a principle after which they distinguish between chromatin segments that are genomically close and those that are genomically far away. For example the chemical composition of different chromatin segments, e.g. through histone modifications, could play a role at this.

In order to validate our model we compared the geometry and especially the mechanical properties, i.e. flexibility and elasticity, of the model chromatids to experimental findings. With our model we obtained objects that matched the shape of mitotic chromosomes and flexibility of chromatids assembled from Xenopus laevis egg extract [23]. In particular, we observed a much increased bending stiffness compared to simple polymer models such as self-avoiding walks, which can be explained by the entropic repulsion between the chromatin loops that are formed by the cross-linking of the fibre. Simulations of applied stretching forces revealed changes in the loop structure with a reorganization for small forces, followed by breakage of loops at large forces. We found that the loop structure, the size and the average number of cross-links within the chromatin fibre are essential for the mechanical properties. Therefore we suggest that altered physical dimensions and mechanical properties in different stages of mitosis and across different species can be explained by different internal loop structures.

At high looping probabilities, the cross-linking of the fibre results in a condensation into a homogeneous, rod-like shape. The lengths and widths of the model chromatids match observations of chromosomes of numerous species, in particular those assembled from Xenopus egg extract [23], [45]. We found that the length and the thickness of the model chromatids are governed by the size restriction for chromatin loops and the number of loops within the chromatin fibre. The linear dependency of the chromatid thickness with the cutoff length is consistent with the simplified assumption of a helical folding of the chromatin fibre, which was in fact one of the earliest propositions for metaphase chromosome structure [9], [22]. However, the Dynamic Loop Model for mitotic chromosomes is not a simple helix but rather resembles a chromatin network with the tendency of the fibre to form rings around the central axes. A chromatin network was considered as the structure of mitotic chromosomes before [25], [30].

In our coarse grained description we obtain lengthwise compaction ratios between 10 and 30 fold of the native length, depending on the upper restriction for the loop size. As the compaction from the 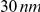 fibre to the mitotic chromosomes is in the range of 500-fold, this would suggest that our coarse-grained chromatin fibre has a diameter much larger than 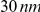 but still well below 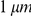. Our model can therefore be seen in the context of a hierarchical folding model for the mitotic chromosome [17], [46]. Here the dynamic formation of cross-links would account for the compaction in one hierarchy level. On the other hand we find that for fixed 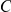-to-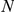 values and fixed 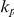, the lengthwise compaction ratio increases with the chain length as the loop structure becomes finer. Therefore the Dynamic Loop Model is able to produce high compactions when the chain length is large enough. The network model that was put forward by Poirier and Marko assumes the cross-linking of the 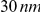 fibre [24]. However, the simulation of such high compaction ratios requires the equilibration of very long polymers that is computationally not viable.

Experimental results suggest that the flexibility of chromosomes is subject to the species and to the stage of mitosis [20]. In our simulations we found ratios of persistence length to thickness of the chromatids in the range of 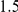 to 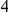, depending of the choice of parameters. This is consistent with the experimental findings of Houchmandzadeh and Dimitrov [23] on Xenopus egg extract who reported a ratio of approx. 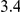. Hence, the bending rigidity of the chromatin structure in the egg extract can be explained by the loop formation alone, without the assumption of a protein scaffold. However, other experiments of in vitro and in vivo assembled chromosomes from Xenopus cells, newt lung cells, the newt TVI cell line and Drosophila cells found much higher bending rigidities with persistence lengths that are many times of the actual chromosome length [27], [28]. Poirier et al [28] suggested that the differences between the egg extract and in vivo assembled chromosomes arise from different chromatin organization in both systems. This could be connected to the different functions of egg extract and somatic tissue culture cells or because egg extract chromatids are not completely condensed. We observed for the Dynamic Loop Model that increasing looping probabilities resulted in much higher bending rigidities, thus supporting the argument that not fully condensed chromosomes are more flexible. Moreover, condensins, which are the main candidates for the binding proteins, were found to be able to dimerize and also to form heterodimers with other proteins [47], [48]. When cross-links can cluster in this way, it has to be assumed that the loop concentrations in real chromosomes are much higher than it is possible to model in our coarse grained approach. Therefore, consideration of such protein-protein interactions in the model could also account for an enhanced stiffness.

Furthermore, we have to point out that the entropic repulsion between chromatin loops is not the only factor that determines the flexibility of chromatids. Rather we suggest that these entropy effects contribute to the bending rigidity, and in some cases, such as for chromatids from Xenopus egg extract, are sufficient to explain them. However, there are certainly other factors, such as the surrounding solvent, that do also contribute to the mechanical properties.

The stretching simulations revealed that the looping mechanism results in a very elastic chromatid that can be stretched to many times of its native length. For elongations of up to three times of the native length, a linear relationship between stretching force and relative extension was found. This is in agreement with experimental findings where chromosomes as well as single chromatids behave like a homogeneous elastic material [22], [24]. Our results show clearly that the number of loops is of great importance for the elastic response of the chromatid. For fixed chain length 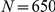 and fixed cutoff length 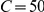 we observed that the increase of the initial average loop concentration from 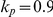 to 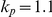 is associated with a doubling of the Young modulus. Experimental evidence for this dependency was given by Almagro et al [7]. The authors measured the elastic response of Xenopus egg extract chromatids after cleavage of SMCs with trypsin. It was found that chromosomal domains containing SMC proteins had a much higher stretching stiffness (up to four times) than domains where parts of these SMC proteins were cleaved. As SMCs are subunits of condensin proteins which are most likely to be responsible for chromatin cross-linking, our results confirm this experimental finding, as higher loop concentrations in the Dynamic Loop Model are also associated with an increased stretching stiffness.

Closer examination of the loop structure in this region of small relative extensions showed that a reorganization takes place when the chromatid is stretched. Such a behaviour was proposed before in the network model of Poirier et al [26]. The loop size distribution shows a shift from large loops to small loops and therefore leads to a thinning of the chromatid. The measurement of values of 0.045 to 0.065 for the Poisson's ratio in our model is in good agreement with experimental results from Poirier et al [24] with a value of 0.069. We have to point out that our Monte Carlo algorithm simulates chromosomes in thermal equilibrium and the stress is introduced by a pulling potential representing the force. However, it might be that this kind of approach does not match experimental conditions as we do not impose a constant stretching rate. Due to the thermal equilibrium situation, the forces in the simulation were much lower than what one would get if the pulling process was assumed to be a non-equilibrium process.

For extensions higher than 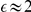, a strong leveling off of the force extension curve occurred, resulting in force plateaus. Force plateaus were also observed in stretching experiments although only for long extensions of 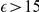 and more. The fact that in our simulations the plateau regions started much earlier can be explained by the coarse grained character of the polymer model. In reality, the structure of the chromosomes are certainly much finer. In addition, the loop concentrations in real chromosomes are most probably also much higher, considering the effect of SMC dimerization and heterodimerization as has been pointed out before. However, at present, simulations of much finer systems are still computationally not feasible. Moreover, our model does not include elasticity of the underlying coiling chromatin fibre itself, which could also contribute to the total elasticity of the whole chromatid [49].

Different chromosome states after retraction from extension into the plateau region were reported for chromosomes from different animals and different ways of assembling (in vivo or in vitro). While Poirier et al [24] observed a swollen ghost state, Houchmandzadeh et al [22], [23] witnessed non homogeneous chromatids with alternating thick and thin regions and which are up to five times longer than originally. Such inhomogeneous chromatids are obtained in the Dynamic Loop Model when the number of loops are small and consequently cross-links are not located homogeneously along the chain (see Figure 1A). A possible reason for this is that binding sites are destroyed when the elongation is too far. Therefore, in the retraction process the looping probability might be much lower than in the original chain which in turn results in the longer and inhomogeneous chromosomes.

We have shown that the dynamic cross-linking mechanism leads to the condensation of the chromatin fibre. The loops within the fibre evoke an increased bending stiffness by entropic repulsive forces. Our model is able to explain the shape of mitotic chromosomes and the flexibility of mitotic chromosomes assembled from Xenopus egg extract. Furthermore, simulations of stretching forces showed good qualitative match of our results with experimental findings. We therefore conclude that the structure and mechanical properties of mitotic chromosomes are in a great part invoked by internal formation of loops of the chromatin fibre.

## Materials and Methods

### Polymer Models

Due to the size and the high complexity of chromatin in the cell nucleus, computer models have to make simplifications in order to remain viable. Coarse grained polymer models have proved to be a good tool to model the chromatin fibre. A polymer consists of 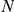 monomers with positions 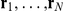. Each monomer is permanently linked to its neighbours by bond vectors 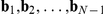. The size of polymers can be described for example by the mean squared end-to-end distance, which often obeys the scaling law
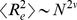
(1)where 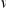 is a model specific scaling exponent. For the ideal chain and the Gaussian chain without excluded volume the exponent is 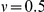. When excluded volume interaction is included, the polymer has the scaling exponent of 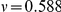.

### Dynamic Loop Model

The main idea of the model is that the tight condensation of the mitotic chromosome, which is presumably facilitated by condensin proteins and Topoisomerase II, can be modeled by a dynamic looping mechanism of the chromatin fibre. The model assumes, that genomically distant sections of the chromatin fibre can cross-link for a fixed amount of time when they come into physical proximity of each other. This self-tethering mechanism mimics the dynamics of binding proteins such as Topoisomerase II and condensins that have been found to be significant for metaphase chromosome structure. Although the exact role of Topoisomerase II and condensins in mitotic chromosomes is still unclear, it is ascertained that they are able to bind to chromatin and to cross-link the fibre [31]. However, the important element in the model is the probabilistic nature of the cross-linking mechanism. Rather than being a fixed structure, the organization of the fibre is dynamic. This accounts for the fact that proteins in the surrounding solvent of the chromatin fibre are mobile. Therefore also the binding sites are subject to fluctuations in space and time, which mirrors the effect of protein concentration and binding affinity.

### Monte Carlo Simulations

The behaviour of the chromatin fibre is simulated using a lattice Monte Carlo algorithm based on the well-established Bond Fluctuation Model (BFM), which incorporates excluded volume interactions and preservation of the topological state of the polymer [50]. The Monte Carlo algorithm for the Dynamic Loop Model consists of two main steps. In the first step, local moves for the single monomers are proposed and accepted if the constraints of the bond vectors are not violated. These local moves make sure that the algorithm produces correct Rouse dynamics for the polymer [51]. The key feature of the Dynamic Loop Model is the ability of the fibre to cross-link with itself, which is comprised in the second step. When two fibre segments come into the proximity of each other by diffusion, there is a certain probability 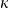 that they form an additional bond between each other and thus a loop in the chromatin fibre. The size of the loops, i.e. the contour length between the bound fibre segments, is restricted by a maximum length 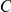. The loop also has a restricted lifetime which is drawn from a Poisson distribution with mean value 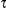. After this lifetime, the cross-link between the fibre segment dissolves.

Conformations were sampled from the equilibrium distribution using the Monte Carlo algorithm described above. The algorithm sweeps the space of possible conformations with equal probability. Simulations were performed on a lattice with periodic boundary conditions so there was no spatial confinement of the polymer. As in all Metropolis Monte Carlo algorithms, subsequent conformations are highly correlated to each other. In order to obtain uncorrelated conformations from the simulation, the autocorrelation time has to be considered. Moreover a certain number of steps are required to reach thermal equilibrium from the start configuration. The integrated autocorrelation time 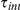 was used in this work to calculate the autocorrelation time. 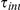 is determined by the autocorrelation function 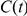 and the normalized autocorrelation function 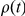 respectively. These functions measure the correlation of a certain observable for conformations which are separated by 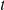 Monte Carlo steps. Let 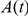 be an observable, then the unnormalized autocorrelation function for 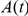 is given by
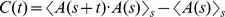
(2)and the normalized autocorrelation function is simply 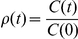, where 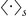 denotes the average over the thermal ensemble at step 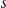. For finite samples the average values can be estimated by mean values. The windowing procedure described in [52] was used to obtain an estimate of the integrated autocorrelation time
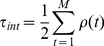
(3)


The integer 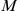 was chosen such that 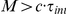. According to Sokal [52], 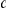 can vary between 4 for exponential decaying 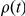 to 10 for slower decay. In this study we used 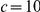 for all simulation runs. Two subsequent conformations are considered to be uncorrelated when more than 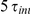 steps are between them. In the beginning of the simulation, 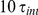 steps are considered to be enough for equilibration of the starting configuration. In our simulations, two such initialization stages were run. In the first stage the looping mechanism was still turned off, the fibre thus equilibrated from the start configuration to a self-avoiding walk. In the second stage, which started after 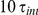, the looping mechanism was switched on and the Dynamic Loop Model simulation started. After again 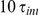 the system was considered to be in thermal equilibrium.

Polymer chains consisted of 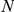 monomers. Simulations with various values for 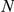 between 400 and 800 were performed. To properly investigate the influence of the cutoff length 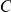 and the number of loops per chain length 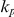, we conducted runs with varying parameters. 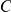 took values between 20 and 80 and for 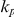 values between 0.5 and 1.4 were considered. Typically, the autocorrelation time for a self-avoiding walk scales with the square of the number of statistical segments 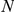. With the high densities in our model chromosomes and consequently the high rejection rates, this made it hard to model longer chains. The exact autocorrelation times in the simulations were also depending on the cutoff length and mean loop concentration. As an example, the autocorrelation time for a configuration with 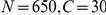 and 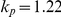 was about 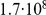 MC steps. Around 5000 independent conformations could be sampled in 96 hours by parallel simulations running on 64 processors.

For the simulation of the stretching of model chromatids a force 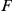 was included via a potential 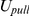. The direction of the force is parallel to the end-to-end vector of the model chromatid, so the fibre can move without spatial constraints. 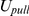 is given by
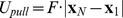
(4)The force 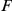 is a parameter in the simulations. The potential 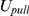 has then the effect that local moves of one of the end monomers, which increase the end-to-end distance, are only accepted with probability 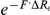. In the stretching simulations the polymers are also in thermal equilibrium. The mean relative extension at force 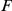 is given by
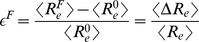
(5)Here 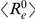 denotes the mean end-to-end distance without any pulling force and 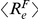 is the mean end-to-end distance for a configuration with forces 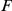.

### Bending rigidity and persistence length

Long polymers usually have bending rigidities that limit their flexibility. While for simple models such as the ideal chain or the Gaussian coil the mean correlation between the bond vectors is 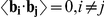, for real polymers this correlation is non-zero. To quantitatively describe the flexibility of polymers the directional correlation of different segments of the polymer can be used. Let 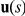 be the direction of a chain segment at the contour length 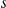. Then the correlation function between two segments separated by the contour length 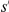 is
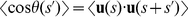
(6)The averaging is done over both, all positions 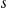 within one conformation and the ensemble of all conformations in thermal equilibrium. A quantity that measures the stiffness of the chain with respect to the orientational correlation is the persistence length 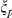. It is defined as the integral width of the correlation function [53]

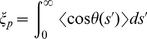
(7)It can be shown that the persistence length is proportional to the bending modulus 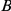 [42]

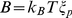
(8)The bending modulus can be described as the quantity which determines how much force is necessary to bend a segment of the chain to a certain curvature. In classical elsticity theory, the bending modulus is connected to Young's modulus 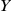 which determines the elastical behaviour of a material. In the case of a homogeneous cylinder with radius 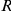, the relationship is
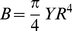
(9)


### Estimating backbones, directional correlation and radial density

In this work the bending stiffness of the model chromatids were estimated via the directional correlation of segments of the chromatids. For this, for each conformation a backbone which represents the alignment of the model chromatid was calculated. The polymer chain which is given by the position vectors of the monomers 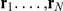 was divided into 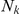 segments of 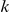 monomers each. The center of masses of these segments 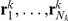 then represented a new, coarse-grained chain that approximated the alignment of the coiled chromatin fibre (Figure 2).

With the imaginary backbone the mean directional correlation between distant segments of the model chromatids were determined. Let 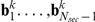 denote the bond vectors of the backbone, where 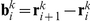. Then the mean directional correlation 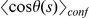 between segments that are separated by the arc length 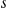 for one single conformation is given by
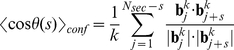
(10)Let 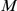 be the sample size of the Monte-Carlo simulation, then the thermal average was estimated by
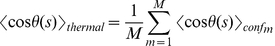
(11)


The directional correlation was used to test if the coarse graining level 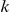 for the calculation of the backbone was chosen correctly. In this case the directional correlation showed a exponential decaying behaviour, whereas for coarse graining levels that were too low or too high, the behaviour would be non-exponential.

The backbone that is determined using the method described above is a coarse grained backbone and suitable to analyse the mean directional correlation between segments of the chromatid. However, for the analysis of other properties such as the thickness of the chromatid, a less coarse grained backbone is more advantageous. Such a nearly smooth backbone is obtained using a similar method. A coarse graining level 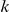 is selected again and the backbone is build in the following way: the first point of the backbone chain is the center of mass of monomers 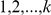, the second point of the backbone is the center of mass of monomers 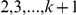 et cetera. Thus, a chain is obtained where the beads are spatially very close to each other and the backbone can be considered as a smooth trajectory. These backbones were used to estimate the length and the thickness of the chromatid by calculating the mean radial density perpendicular to the backbone. The chromosome radial thickness 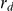 was estimated as the distance for which 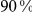 of all monomers were aligned closer to the backbone than this distance.

To estimate the thickness of model chromatids under an external force, backbones were calculated using the same coarse graining level as for the model chromatids without stretching force. As the total number of loops in the linear elongation region only changes marginally, it is justified to assume that the same coarse graining level yields the correct backbone.
